# Photodynamic Therapy for Gynecological Diseases and Breast Cancer

**DOI:** 10.3969/j.issn.2095-3941.2012.01.002

**Published:** 2012-03

**Authors:** Natashis Shishkova, Olga Kuznetsova, Temirbolat Berezov

**Affiliations:** Department of Biochemistry, School of Medicine, People’s Friendship University of Russia, Moscow 117198, Russia

**Keywords:** photodynamic therapy, photosensitizers, cervical/vulvar intraepithelial neoplasia, ovarian neoplasms, breast neoplasms

## Abstract

Photodynamic therapy (PDT) is a minimally invasive and promising new method in cancer treatment. Cytotoxic reactive oxygen species (ROS) are generated by the tissue-localized non-toxic sensitizer upon illumination and in the presence of oxygen. Thus, selective destruction of a targeted tumor may be achieved. Compared with traditional cancer treatment, PDI has advantages including higher selectivity and lower rate of toxicity. The high degree of selectivity of the proposed method was applied to cancer diagnosis using fluorescence. This article reviews previous studies done on PDT treatment and photodetection of cervical intraepithelial neoplasia, vulvar intraepithelial neoplasia, ovarian and breast cancer, and PDT application in treating non-cancer lesions. The article also highlights the clinical responses to PDT, and discusses the possibility of enhancing treatment efficacy by combination with immunotherapy and targeted therapy.

## Introduction

**Figure 1:**
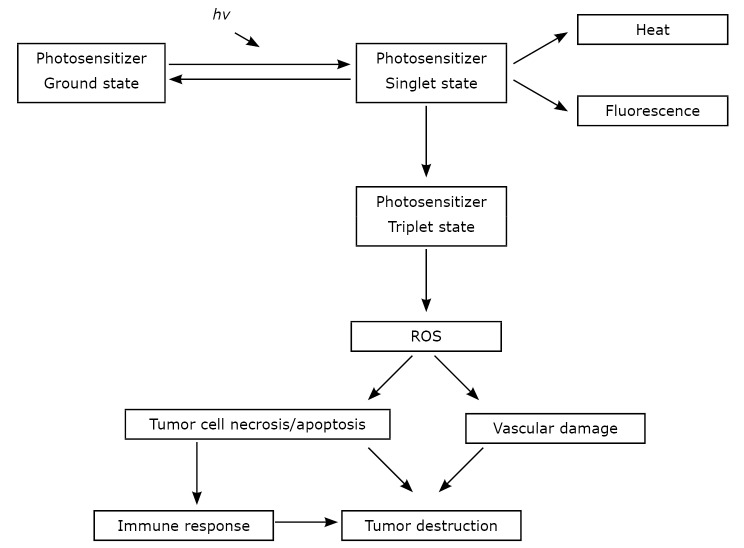
Photodynamic therapy mechanism, as explained in detail in the text.

Photodynamic therapy (PDT) is a mode of therapy used in cancer treatment where drug activity is locally controlled by light (**Figure 1**). The ground state non-toxic photosensitizer achieves a higher unstable energy state (singlet state) upon illumination with an appropriate light wavelength and in the presence of oxygen. In this unstable state, the activated photosensitizer releases energy either by emitting heat and light or by the conversion of the unstable state into an intermediate energy state (triplet state) before returning to the stable ground state. In the triplet state, the photosensitizer generates reactive oxygen species (ROS), such as superoxide and hydroxyl radicals or singlet oxygen. ROS rapidly reacts with biological substrates, which initiates an apoptotic or necrotic response. This process eventually leads to oxidative damage and cell death. The intracellular localization of the photosensitizer activity is of great importance because mitochondrial damage generally leads to apoptosis, whereas plasma membrane damage induces necrosis ^[^^1^^-^^3^^]^. Photofrin, one of the most widely used photosensitizers, is localized in the mitochondria due to its hydrophobicity and its affinity to the binding site on the mitochondrial membrane ^[^^4^^]^. A frequently used drug in PDT is 5-aminolaevulinic acid (ALA). However, 5-ALA is not a photosensitizer, but a precursor of the endogenous photosensitizer protoporphyrin IX, which is a member of the heme synthesis pathway that occurs in the mitochondria ^[^^5^^]^. PDT affects the tumor vasculature, where illumination and ROS production cause vessel shutdown and lead to tumor hypoxia ^[^^6^^]^. PDT also affects the immune system ^[^^7^^, ^^8^^]^. The tissue selectivity of different photosensitizers is currently under investigation. Moreover, the laser irradiation restricted within the lesion area combined with the short lifespan of the emerging cytotoxic species ensures that phototoxic damage is mainly localized in the lesion with minimal inclusion of the surrounding tissues. The application mode of photosensitizers may be topical or systemic (oral or intravenous). Indications for PDT include cancers where the entire lesion is visible through an endoscope to enable laser irradiation. PDT has been applied for the treatment of skin cancer, surperficial esophageal cancer, lung cancer, and gastric cancer ^[^^9^^, ^^10^^]^. In addition, bladder and prostate cancers have been treated with PDT ^[^^11^^–^^13^^]^. In this review, PDT effectiveness in treating gynecological cancers, such as ovarian cancer, cervical intraepithelial neoplasia (CIN), and vulvar intraepithelial neoplasia (VIN) is discussed. The available data on targeted and non-targeted treatment modes for breast cancer is also summarized.

## Cervical Intraepithelial Neoplasia (CIN)

Cervical cancer is the second most common malignancy in women worldwide, with about 500,000 new patients diagnosed every year. CIN is a precancerous condition localized in the squamo-columnar junction of the cervix uteri. The classification ranges from CIN 1 (mild dysplasia) to CIN 3 (severe dysplasia). Human papillomavirus (HPV) infection, mainly high-risk types 16 and 18, is directly related to the development of CIN and cancer ^[^^14^^]^. CIN prevalence has increased during the last few decades particularly among younger women. Results of recently established screening programs showed that the incidence of cervical cancer has decreased by about 50% to 60%. The recent standard of care consists of excision, e.g., loop electrosurgical excision procedure (LEEP), cold knife excision of the transformation zone or local destruction by laser or cryotherapy ^[^^15^^]^. These procedures are commonly painful during treatment and may cause post-operative bleeding. Thus, anesthesia is usually used as a necessary prerogative. The recurrence rates of CIN lesions for more than six months after the treatment are between 5% and 16% for LEEP, 4% and 24% for cryotherapy, and 3% and 30% for laser therapy ^[^^16^^]^. The major drawback of these excision methods is the destruction of the cervical stroma, which may cause cervical insufficiency and/or cervical scar structure that may lead to premature delivery and increase the risk of infertility and the need for caesarean operation ^[^^17^^–^^19^^]^.

## PDT in CIN Treatment

PDT has been used as an alternative therapy to meet the demands of the increasing number of younger patients waiting to preserve fertility and to accommodate high-risk patients and those who refuse surgery. PDT can be performed without anesthesia because the patient experiences no pain during the procedure. Furthermore, bleeding does not occur during PDT, and fertility can be preserved while leaving the cervix relatively intact because no adverse effects of PDT have been noted on pregnancy and delivery. In a previous study, 22 patients became pregnant after receiving PDT with Photofrin and 12 of them went on to have normal transvaginal deliveries ^[^^20^^]^. The same study also reported on PDT with Photofrin on 131 cases (95 CIN, 31 dysplasia, 1 VIN, 3 squamous cell carcinoma, and 1 endocervical adenocarcinoma) ^[^^21^^]^. Of these cases, 127 responded favorably to the treatment (96.9%). However, the main side effect of PDT with Photofrin was skin photosensitivity ^[^^22^^]^. Thus, PDT with topically applied photosensitizers, mainly 5-ALA and its esterified derivate hexylaminolevulinate (HAL), was used^[^^23^^–^^29^^]^. A cylindrical light applicator with a backscattering surface for light homogenization was used to illuminate the endocervical canal. A tip, which reached the endocervical canal, fixed the light applicator to the cervix and provided endocervical illumination. From a clinical point of view, the application of liquid solutions to the cervix could be problematic. Three studies have shown that the application of 5 mL to 10 mL of 10% solution of 5-ALA to the portio uteri using an appropriately sized cervical cap for 4 h to 8 h followed by illumination using a laser (wavelength 635 nm) through a specially developed cervical applicator induces regression of the CIN lesion, but does not entirely eradicate the lesion ^[^^23^^-^^25^^]^. However, PDT with the application of 12% of ALA was very effective in eradicating 80% of cervical HPV infection ^[^^26^^, ^^27^^]^. The application of a special thermogel was performed to improve the adhesion of the 5-ALA solution ^[^^28^^]^. The application of 5-ALA gel followed by laser illumination using a special light catheter led to a complete response in 4 out of 5 patients suffering from CIN 2 for 9 months ^[^^29^^]^.

Promising results have been demonstrated with HAL, which is an esterified derivate of 5-ALA, in PDT ^[^^30^^]^. Topically applied HAL had a high epithelial fluorescence as a result of significant selectivity of epithelium over the lamina propria ^[^^31^^, ^^32^^]^. PDT with HAL thermogel applied to the cervix followed by laser illumination (wavelength 633 nm) resulted in a complete response and remission of HPV infection for 6 months in 15 out of 24 patients with CIN ^[^^33^^]^. The remission rates were 71%, 50%, and 71% for CIN 1, 2, and 3, respectively. By comparison, the topical treatment with recombinant human interferon gamma resulted in the complete regression of CIN and remission of HPV infection in only 53% of the treated cases ^[^^34^^]^.

Therefore, HAL has been suggested as a promising molecule for both selective fluorescence diagnosis and PDT treatment of precancerous and cancerous epithelial lesions. Moreover, the topical application of the photosensitizer is of great advantage because it leads to less systemic exposure, as well as rapid clearance without causing cutaneous photosensitivity.

## Vulvar Intraepithelial Neoplasia (VIN)

Vulvar intraepithelial neoplasia (VIN) is a precancerous skin lesion of the vulva, and is described as the dysplasia of squamous epithelium of the vulva. VIN is not an invasive cancer, but may eventually become an invasive squamous cell cancer if left untreated. Before 2004, VIN was classified from VIN 1 (mild atypia) to VIN 3 (severe atypia). However, the International Society for the Vulvovaginal Diseases has reclassified VIN. The term VIN now refers only to high-grade abnormal squamous lesions (previously known as VIN 2 and VIN 3). VIN is most commonly seen in immunocompromised and postmenopausal women ^[^^35^^]^. The current incidence of VIN 3 is increasing, whereas the mean age of affected women is decreasing. About 28% to 52% of patients with VIN 3 are aged 40 years or younger ^[^^36^^]^. The risk factors are similar to those with CIN. Race, parity, and comorbid medical conditions seem to have no role in VIN development, whereas cigarette smoking is believed associated with VIN development ^[^^37^^]^. Moreover, an etiological relationship exists between VIN development and 80% to 90% of HPV type 16 infection cases^[^^37^^]^. The symptoms include itching or burning associated with vulvar irritation, dyspareunia, and labial erythema or swelling. Asymptomatic lesions are less common, and spontaneous regression occurs in less than 1.5% cases ^[^^38^^]^. The traditional treatment options of surgical excision and CO_2_ laser ablation are both associated with high rates of disease recurrence, particularly with multifocal disease ^[^^39^^]^.

## PDT in VIN Treatment

The treatment of VIN with PDT is currently in an investigational procedure. Most published studies have used local application of 5-ALA. Martin-Hirsch et al. ^[^^40^^]^ reported that 16 out of 18 patients who received PDT had symptom relief (89%), and 9 out of 10 patients developed local recurrence at the treatment site detected during the follow up procedure 2 years after the treatment. In a subsequent study of PDT treatment, 5 out of 6 women suffered from a persistent disease at the follow-up visit 6 months after the treatment ^[^^41^^]^. In yet another study, 25 women with 111 lesions of VIN 2 and VIN 3 were topically sensitized with 10 mL of 20% solution of 5-ALA, and were treated with 57 cycles of 100 J/cm^2^ laser-light at 635 nm ^[^^42^^]^. Overall, 64% of the 111 lesions regressed after various PDT cycles, and a complete histological response was achieved in 52% of the lesions. The hyperpigmented and hyperkeratotic patches of VIN were the least responsive to the treatment. Depending on the type of lesion, the recommended treatment included scraping off the upper keratinized cell layer with a lancet or ring curette to improve the penetration of the photosensitizer ^[^^43^^]^. A previous study used mTHPC for PDT treatment of VIN ^[^^44^^]^. In this study, all 6 patients suffered from edema and ulceration with slough formation a week after the intravenous application of PDT, and 4 of these patients showed a full response to the treatment at the follow-up medical appointment 6 months after the treatment. Although mTHPC-PDT appears to be a very effective treatment, its major drawback is light photosensitivity for one to 2 weeks after treatment ^[^^44^^]^.

PDT represents an alternative treatment method for VIN that is easy to perform and has the advantages of minimal destruction of healthy tissue, a shorter healing-time, and excellent cosmetic results as opposed to the standard of care procedures.

A combination of immunotherapy with imiquimod and methylated ALA- PDT was applied to achieve better results in VIN treatment. The rationale behind the use of the combination treatment was that a short amount of imiquimod should stimulate a local immune response that would enhance the effect of PDT. A clinical response rate of 60% in 32 women with VIN 3 was observed at the 52-week time point^[^^45^^]^. A combination of PDT with HPV vaccines was suggested for patients with persistent HPV infection. Park et al. ^[^^46^^]^ have reported that in tumor models, a combination of PDT and interleukin-12 (IL-12), which stimulates the cytolytic activity of T-cells, NK cell, and lymphokine activated killer (LAK) cells, significantly augmented antitumor effects. However, the combination of PDT and HPV-specific immunotherapy merits further evaluation in clinical trials.

PDT has been successfully evaluated in HPV-associated lower genital tract dysplasia, such as CIN and VIN. In studies on VIN, photosensitizers have been used in various forms, namely, creams, gels, solutions, bio-adhesive patches, and injections. However, a major disadvantage of systemic photosensitizing agents is the prolonged generalized photosensitivity lasting for one or two weeks, which requires a strict light-protection regime. Therefore, developing optimized and standardized methods of photosensitizer delivery to the vulva and cervix is of great importance.

## Ovarian Cancer

Carcinoma of the ovary is one of the most common gynecological malignancies, and is the fourth most frequent cause of fatality in women in the United States ^[^^47^^]^. The most common type of ovarian cancer is epithelial carcinoma. The tumors with low malignant potential, which are also known as borderline tumors, are the most well-differentiated (Grade 0), and account for 15% of all epithelial carcinomas in the ovary. The other three grades are well-differentiated (Grade I), moderately-differentiated (Grade II), and poorly-differentiated (Grade III/IV). Well-differentiated tumors have a better prognosis than poorly-differentiated ones. Clear cell carcinoma, especially undifferentiated carcinoma, has a poorer prognosis than the other cell types. Ovarian cancer spreads early by shedding malignant cells into the abdominal cavity and pelvic, aortic, groin, and neck lymph nodes. Many women with early stages of ovarian carcinoma do not suffer from any symptoms. Unfortunately, two-thirds of all women with ovarian carcinoma have advanced disease at the time of diagnosis. Many women are only diagnosed when they have developed abdominal distention because of ascites, although some cases are diagnosed during a routine gynecologic examination.

The primary treatment modalities are maximal cytoreductive surgical procedures such as adnectomy, hysterectomy, omentectomy, and pelvic limpadenectomy. In less than 20% of patients with stage III/IV malignancy, bowl resection is necessary to achieve optimal tumor reduction at the time of primary surgery. Peritoneal debulking is often necessary because of the local intraabdominal shedding of tumor cells and tumor growth on peritoneal surfaces ^[^^48^^]^. However, most of these patients experience local progression or recurrence even after aggressive cytoreductive surgery and platinum-based chemotherapy. Moreover, less than 17% patients with stage IV disease and 30% patients with stage III ovarian carcinoma will survive for more than 5 years ^[^^49^^]^. The reason for treatment failure is probably related with the difficulties inherent in surgery within the peritoneal cavity, problems related to cytotoxic agent administration to the tumor cells in cytotoxic concentrations, and the ability of ovarian cancer cells to develop resistance against standard chemotherapies. Patients having platinum-resistant ovarian carcinoma with poor prognosis might benefit from strategies that use a fluorescence staining procedure because of its high selectivity in detecting minimal tumor residuals.

## PDT in Ovarian Carcinoma

The use of fluorescence-based visualization techniques to overcome poor diagnostic efficiency on the large surface area of the human peritoneal cavity might acquire greater importance in the future. Fluorescence photodetection takes advantage of the optical properties of tissues, either inherent or induced by exogenously administered photoactive compounds. Cancerous tissue often displays differences in the resulting optical characteristics. Fluorescence imaging after ALA application can distinguish normal from malignant tissue, as was previously shown by Kriegmair et al. for early bladder cancer ^[^^50^^]^. In contrast to conventional white light conditions, ALA-induced fluorescence diagnosis allows significant improvements in the detection of very small tumor lesions, as well as of occult tumors. The application of 5-ALA for fluorescence-guided second-look laparoscopy has been shown to be a promising new procedure in the early diagnosis of ovarian carcinoma metastasis ^[^^51^^, ^^52^^]^. Thirty-six women with ovarian carcinoma and two women with fallopian tube cancer underwent a second-look laparoscopic procedure after receiving an intraperitoneally application of ALA, which is a precursor of the potent photosensitizer protoporphyrin IX (PPIX) ^[^^52^^]^. From a subgroup of 17 patients, 36 tissue samples were obtained from fluorescent and non-fluorescent areas for fluorescence microscopy. The results from the fluorescence microscopy showed that PPIX distribution is strongly concentrated in the tumor tissue in ovarian carcinoma metastases. A specificity of 88% and sensitivity of 100% were calculated for the applicability of PPIX fluorescence in detecting tumor tissues. Similar results were obtained also from animals through the intraperitoneal administration of HAL ^[^^53^^]^. HAL has been shown to produce higher PPIX fluorescence than its parent substance, ALA, at the same concentration and application time and, hence it has been suggested that HAL may replace ALA in topical applications. Photodiagnostic techniques, such as the use of ALA or HAL in detecting occult ovarian cancer tumors, provide a platform technology that permits minimally intrusive investigation, allow detection by endoscopy, and eventually eliminate ovarian cancer cells at their earliest stages.

PDT with methyl-ALA was recently applied for various types of human ovarian cancer in a subcutaneous xenograft model in nude mice ^[^^54^^]^. Methyl-ALA-PDT significantly increased apoptosis and reduced angiogenesis in implanted tumor. Thus, it was concluded that methyl-ALA PDT could be an effective treatment in ovarian cancer, and has a potential application in treating intraperitoneally disseminated micro-foci during surgery. The effectiveness of PDT with Photofrin was evaluated in Phase II clinical trials on ovarian cancer patients ^[^^55^^]^. However, only 2 of the 13 ovarian carcinoma patients treated in a similar trial did not have a recurrence^[^^56^^]^. The most common site of recurrence was the pelvis. A combined therapy was suggested to overcome recurrence and to enhance the PDT efficacy. Meanwhile, ovarian carcinoma cells have been found to overexpress epidermal growth factor receptor (EGFR), and EGFR overexpression has been associated with pure clinical outcomes ^[^^57^^]^. This data provides a strong rationale for the use of inhibitors of EGFR signaling in ovarian carcinoma. del Carmen et al. ^[^^58^^]^ have found that intraperitoneal administration of C225, humanized murine monoclonal antibody against EGFR, and benzoporphyrin derivative monoacid-A (BPD)-based PDT synergistically act in a murine model of peritoneally disseminated ovarian carcinoma. The present study has proven that the combination of these two alternative methods might improve results obtained from Photofrin PDT in treating ovarian carcinoma patients.

## PDT for Noncancer Lesions

### PDT for extrauterine pregnancy

Many patients with extrauterine pregnancy (EUP) require conservative or radical surgery. The main risk with conservative surgery lies on the incomplete placental removal and persistent diseases that require further surgery ^[^^59^^]^. Radical surgery, although effective, usually impairs fertility^[^^60^^]^. This limitation implies that novel treatment options must be developed, and one possible approach is PDT. Only two reports on photoablating rat pregnancies have been found. Yang et al. ^[^^61^^]^ have used the systemic administration of 5-ALA while illuminating the entire uterine horn in pregnant rats. This resulted in massive endometrial ablation and the loss of all embryos in the treated horn. Indiscriminate embryo removal in all pregnancies resulted in high infertility rates. Only 66.2% of the animals that underwent implantation in the treated horn became pregnant, but with 28% fewer embryos per litter. Glinert et al. ^[^^62^^]^ applied direct placental injection of a Palladium-bacteriochlorophyll derivative, and illumination was performed to achieve selective photoablation . Nearly 80% of the treated rat embryos were selectively photoablated, which left the remaining litter unharmed to achieve normal parturition. The treated animals retained their fertility and developed normal implants in both treated and untreated uterine horns. Thus, PDT is a promising treatment method in clinical treatment of EUP. However, further studies are still needed to evaluate the potential tubal damage and local/systemic adverse tissue reaction before applying PDT to humans.

### PDT for endometriosis

Endometriosis is defined as the presence of endometrial-like tissues outside the uterus. These tissues induce chronic inflammatory reaction, scar, and adhesions that may distort the pelvic anatomy of a woman ^[^^63^^, ^^64^^]^. Endometriosis is a very common debilitating disease that occurs in 6% to 10% of the general female population. In women who experience pelvic pains, infertility, or both, the frequency of endometriosis is between 35% and 50% ^[^^65^^]^. Patients with endometriosis mainly complain of pelvic pain, dysmenorrhea, and dyspareunia^[^^66^^]^. Thus, therapies focus on relief of symptoms, resolution of existing endometriotic implants, and prevention of new ectopic endometrial tissue foci. Current therapeutic approaches are far from being curative because they focus only on managing the clinical symptoms rather than fighting the disease. A combination of surgical treatment and medical therapy has been suggested for treating endometriosis to enhance fertility. However, no studies have demonstrated significant efficacies ^[^^67^^–^^69^^]^. Yang et al. ^[^^70^^]^ used 5-ALA for the photodetection of experimentally induced endometriosis in a rat model, and found that the fluorescence intensity of PPIX was significantly greater in implants than in adjacent normal peritoneum. This result shows that a potential new approach can be developed to diagnose and treat endometriosis. On the other hand, Krzemien et al. ^[^^71^^]^ compared the effects of ALA-PDT, electrosurgery, and surgical resection for endometriotic explant ablation in a rat endometriosis model . They found that ALA-PDT resulted in ablation of endometriotic explants with no adhesions, in contrast to electrosurgery and surgical resection, which resulted in a greater incidence of surface adhesions. This result suggests the potential application of ALA-PDT as a distinct therapeutic treatment compared with conventional endometriosis treatments.

## PDT for Breast Cancer

Approximately 5% to 19% of breast cancer patients suffer from chest wall recurrences after mastectomy ^[^^72^^]^. Breast cancer frequently recurs in the skin and soft tissues of the chest wall. These tumors usually involve relatively large areas that form large tumors, numerous small nodules or infiltrating sheets of cells, which cause pain, ulceration, necrosis, and serious skin infection. Surgical removal, site-specific radiotherapy, or both, are the common treatment procedures for chest wall metastasis ^[^^73^^, ^^74^^]^. About half of the patients do not experience adequate tumor control with these therapies. Meanwhile, PDT has been experimentally used to treat recurrent breast cancers in the chest wall. A few clinical trials on PDT have been performed to treat chest wall metastasis. A phase I clinical study of PDT using mono-L-aspartyl chlorin e6 (Npe6) as a photosensitizing agent was performed over an 18-month period in the US ^[^^75^^]^. The design of this study consists of a single escalating dose of Npe6 on 11 patients with a variety of solid tumors, including 4 patients with recurrent breast adenocarcinoma. Complete tumor response was observed in 2 out of 4 patients with breast adenocarcinoma. The common adverse effects were pain, erythema, and edema within and adjacent to the treated areas. The use of low-dose Photofrin-induced PDT was suggested to treat chest wall progression of breast carcinoma^[^^76^^, ^^77^^]^. Fourteen patients with more than 500 truncal metastasis were treated with Photofrin-PDT. All patients demonstrated tumor necrosis, with 9 out of 14 complete responses ^[^^76^^]^. Moreover, PDT with m-THPC resulted in a complete response in all 7 patients with breast cancer recurrences ^[^^78^^]^.

## Targeted PDT for Breast Cancer

A new approach for targeted PDT was suggested by Stuchinskaya et al. ^[^^79^^]^. Gold nanoparticles conjugated with antibodies are effective in targeting and possibly destroying cancerous tissue through photothermal reaction ^[^^80^^, ^^81^^]^. A 4-component anti-HER2 antibodies-phthalocyanine-polyethylene glycol-gold nanoparticle conjugate is used as a potential drug for targeted PDT. Cellular experiments have demonstrated that nanoparticle conjugates selectively target breast cancer cells that overexpress the HER2 epidermal growth factor cell surface receptor and are also effective PDT agents. The results of a pilot clinical trial of late-stage breast cancer patients treated via laser immunotherapy (LIT) have been previously presented by Li et al. ^[^^82^^]^ The protocol consisted of three major components, namely, near-infrared laser for non-invasive irradiation, indocyanine green for selective thermal effect, and immunoadjuvant (glycated chitosan) for immunological stimulation. In 8 breast cancer patients with confirmed stage III or IV cancer available for evaluation, the objective response rate was 62.5% and the clinical beneficial response rate was 75%. This preliminary data suggests that LIT, as a new approach that uses the host immune system to fight cancer cells, has a high level of tolerance and is a promising treatment for metastatic breast cancer.

Hu et al. ^[^^83^^, ^^84^^]^ have recently developed a successful targeted PDT that can simultaneously target both tumor neovasculature and tumor cells. The preference of a target molecule as receptor tissue factor (TF) was based on a previous finding that TF was selectively expressed in cells and vascular endothelial cells in 80% to 100% of breast tumors, including multidrug resistant tumors ^[^^85^^, ^^86^^]^. TF is also over-expressed by ovarian cancer cells and many other types of cancer (lung, prostate, and colorectal) ^[^^87^^]^. The conjugates of factor VII (fVII) as ligand for TF with Verteporfin or SnCe6 as photosensitizers were used for the targeted PDT. The study showed that TF-targeting PDT using fVII-conjugates enhanced the effect of non-targeted PDT *in vitro*, and was effective in treating human and murine breast tumor in mice, including chemoresistant breast tumor ^[^^83^^, ^^86^^]^.

## Conclusion

Photodynamic therapy provides an emerging alternative to the standard of care methods in anticancer therapy. PDT has been successfully evaluated in HPV-related genital dysplasia, such as CIN and VIN, in ovarian cancer and in chest wall recurrences of breast cancer (**Table 1**). The preservation of fertility is very important in CIN. The major disadvantage common to all the current standard treatments is the destruction of the cervical stroma, which may cause cervical insufficiency. PDT can be a non-invasive treatment method that preserves cervical function and can easily be performed on an outpatient basis.

**Table 1:** Efficacy of PDT for different types of cancer.

Cancer	Photosensitizer	Response	Cure rate	Publication
	Photofrin	96.6% (127/131)	96.6%	Muroya^21^
		42% (3/7)	42% (3/7)	Hillemanns ^23^
		51% (16/31)	31% (10/31)	Keefe^24^
CIN	5-ALA	75% (9/12)	33% (4/12)	Barnett^25^
		95% (19/20)		Wierrani ^26^
			91% (10/11)	Borner^27^
		80% (4/5)	80%	Wang^29^
	HAL	63% (15/24)	63%	Soergel^33^
		89%	10% (1/10)	Martin-Hirsh^40^
	5-ALA	83%	16% (1/6)	Kurwa^41^
VIN		64%	52% (13/25)	Hillemanns^42^
	mTHPC		66% (4/6)	Cambell^44^
Ovarian	Photofrin		15% (2/13)	Wilson^56^
	Photofrin II	64% (24/37)	13.5% (15/37)	Khan^77^
Chest wall breast cancer recurrences	Photofrin	100% (14/14)	64% (9/14)	Cuenca^76^
	mTHPC	100% (7/7)		Wyss^78^
	Npe6	75% (3/4)	50% (2/4)	Taber^75^

Response means complete ablation or decrease of lesion site. Cure rate means no recurrence of the disease was noted at follow up of 6 months or longer.

For patients with certain types of cancer, such as ovarian cancer, characterized by intraperitoneal dissemination or breast cancer metastasized into the chest wall, effective treatment options are extremely limited. Therefore, using 5-ALA or HAL as photosensitizers provides a platform technology that permits simultaneous detection, diagnosis, and treatment to enable effective administration in a single seamless process. One of the major side effects of PDT is cutaneous phototoxicity, which is dependent on the type of photosensitizer. Therefore, the search for a new photosensitizer with minimal side effects and high selectivity will lead to the development of new methods to diagnose and treat malignant diseases. Moreover, the enhancement of PDT efficacy can be achieved by a combination of immunotherapy or targeted therapy.
